# Genome-Wide Mapping of Transcriptional Regulation and Metabolism Describes Information-Processing Units in *Escherichia coli*

**DOI:** 10.3389/fmicb.2017.01466

**Published:** 2017-08-03

**Authors:** Daniela Ledezma-Tejeida, Cecilia Ishida, Julio Collado-Vides

**Affiliations:** Programa de Genómica Computacional, Centro de Ciencias Genómicas, Universidad Nacional Autónoma de México Cuernavaca, Mexico

**Keywords:** data integration, networks, transcriptional regulation, effector prediction, metabolism, genotype-to-phenotype mapping, information flow

## Abstract

In the face of changes in their environment, bacteria adjust gene expression levels and produce appropriate responses. The individual layers of this process have been widely studied: the transcriptional regulatory network describes the regulatory interactions that produce changes in the metabolic network, both of which are coordinated by the signaling network, but the interplay between them has never been described in a systematic fashion. Here, we formalize the process of detection and processing of environmental information mediated by individual transcription factors (TFs), utilizing a concept termed genetic sensory response units (GENSOR units), which are composed of four components: (1) a signal, (2) signal transduction, (3) genetic switch, and (4) a response. We used experimentally validated data sets from two databases to assemble a GENSOR unit for each of the 189 local TFs of *Escherichia coli* K-12 contained in the RegulonDB database. Further analysis suggested that feedback is a common occurrence in signal processing, and there is a gradient of functional complexity in the response mediated by each TF, as opposed to a one regulator/one pathway rule. Finally, we provide examples of other GENSOR unit applications, such as hypothesis generation, detailed description of cellular decision making, and elucidation of indirect regulatory mechanisms.

## Introduction

Jacob and Monod outlined the relevance of coupling between regulation and metabolism in their discovery of transcriptional regulators (Pardee et al., 1959). They discovered LacI, a protein later termed a transcription factor (TF), that binds to the *lac* operon promoter and represses its expression unless lactose is available. Their model of regulatory activity stated that TFs bind to signaling molecules called effectors, which promote changes in the expression of genes involved in the processing of said molecules. They explained how the cell efficiently manages its resources by only producing specific enzymes when environmental conditions make them necessary, and this is still considered the paradigm for transcriptional regulation: co-regulated genes are assumed to be involved in the same biological process.

Currently, 189 local TFs are listed in RegulonDB, the largest database of transcriptional regulation in *Escherichia coli* K-12 (Gama-Castro et al., 2016), but a genome-scale description of the functional effects of their regulatory activities is still lacking. Previous formalisms have analyzed properties of gene regulation through genetic circuits. Efforts have spanned dynamical modeling (Thomas and D’Ari, 1990) to identifying general network properties (Savageau, 1976, 2001; Kauffman, 1993; Gerosa and Sauer, 2011), but none of them has studied the complete set of regulatory interactions in their functional context.

In the current genomic era, in which “we are drowning in information but starved for knowledge” (Naisbitt, 1984), there is a need for concepts that (a) integrate numerous and different types of molecules and their interactions, (b) reflect biological properties of the cooperation between elements, and (c) can be applied on a small or large scale (Hyduke and Palsson, 2010). Great strides have been made in network analysis to understand how cellular behavior arises from interacting molecules (Ravasz et al., 2002; Shen-Orr et al., 2002; Balazsi et al., 2005). However, the best-studied networks tend to focus on individual layers of interactions, such as TF–gene interactions (Gama-Castro et al., 2016), metabolic reactions (Forster et al., 2003), and signaling pathways (Papin and Palsson, 2004; Papin et al., 2005), which portray an incomplete vision of the information that promotes phenotypes. The goal is to integrate different layers and obtain a thorough picture of the way that functions emerge from combinations of individual mechanisms. This poses a methodological challenge, since some networks are compact and detailed (Alon et al., 1999; Berthoumieux et al., 2014) and others are large and less precise (Karr et al., 2012; Brooks et al., 2014), making it difficult to integrate this information into a single framework that can be used to generate new knowledge. Functional descriptions of the integration also require conceptual improvements. Gene ontologies (GOs) have been the reference for gene classification into biological processes for the past 16 years, but they were conceived to describe individual components rather than the interactions among them (The Gene Ontology Consortium, 2000).

Here, we formalized the process of signal detection to the outset of a functional response, mediated by an individual TF, into four components: (1) signal, (2) conversion of signal into the effector, (3) genetic switch, and (4) response. The integration product of the four components is termed a genetic sensory response unit (GENSOR unit). Ideally, GENSOR units describe the information that flows through different layers of cellular organization to produce an appropriate response (Supplementary Figure S1). We assembled a GENSOR unit for each of the 189 local TFs present in RegulonDB by integrating experimentally validated data from the literature using simple regulons as starting points. Further analysis of the GENSOR unit set showed that less than a quarter of the TFs regulate genes that belong to the same metabolic flux, but feedback is a common occurrence. A gradient of response complexity can be observed and is partially explained by the regulatory effect of the corresponding TF. Beyond the biological insights presented here, we provide the set of GENSOR units as a standardized framework for small- and large-scale analyses of the interplay between transcriptional regulation and metabolism. Last, we show examples of practical applications, such as hypothesis generation, detailed description of cellular decision making, and elucidation of indirect regulatory mechanisms.

## Results

### GENSOR Units of Local TFs in *E. coli* K-12

**FIGURE 1:**
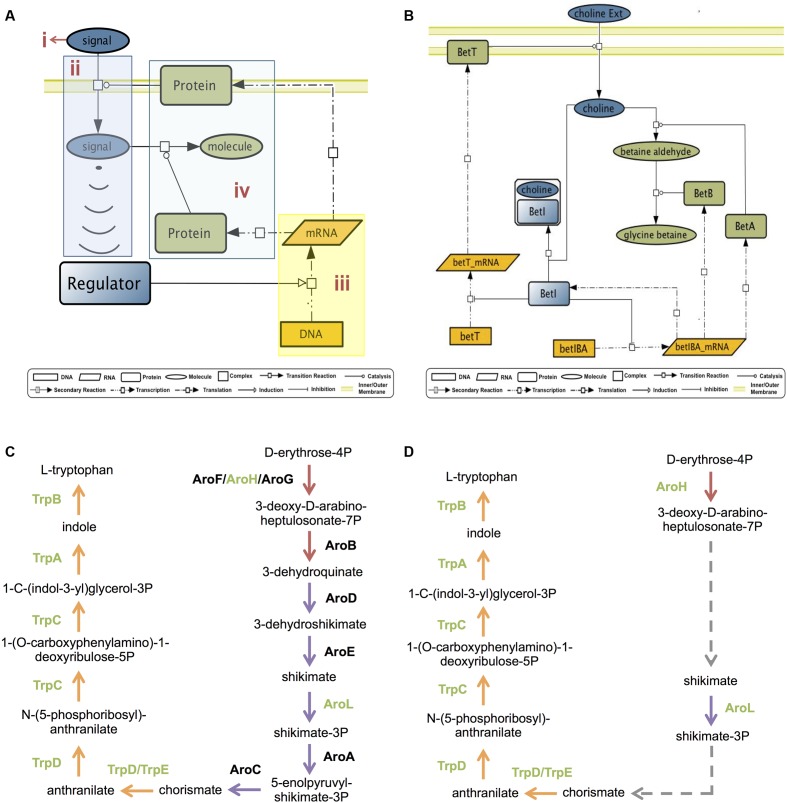
GENSOR units. **(A)** The general GENSOR unit concept includes four components: (i) signal, (ii) signal transduction (shown in blue), (iii) a genetic switch (shown in yellow), and (iv) the response (shown in green). The concept can be applied to any regulator that produces a genetic switch. **(B)** The BetI GENSOR unit. This is an example of a GENSOR unit based on a bacterial TF; color coding for components is as depicted for panel **(A)**. Signal transduction involves transport of choline through the membrane, a genetic switch causes the repression of two transcription units, *betT* and *betIBA*, and the response involves the transformation of choline into glycine betaine. From the higher perspective provided, it is possible to infer that mechanistically in presence of choline, BetI will cease to repress the necessary enzymes for its transport and transformation into glycine betaine. Physiologically, BetI negatively regulates the expression of genes involved in the response to osmotic stress in the absence of choline. The presence of choline induces the expression of the genes required for transport and conversion of choline to glycine betaine, an osmoprotectant. **(C)** Pathways involved in production of L-tryptophan from D-erythrose-4P. The 3-dehydroquinate biosynthesis I pathway is shown in red; the chorismate biosynthesis from 3-dehydroquinate pathway is shown in purple; the L-tryptophan biosynthesis pathway is shown in orange. Enzymes that catalyze reactions are shown beside the reactions. Enzymes regulated by TrpR are shown in green. **(D)** In the TrpR GENSOR unit, the three reactions catalyzed by AroB, AroD, and AroE are summarized in one secondary reaction without indication of the involved enzymes or intermediate metabolites. Another secondary reaction includes reactions catalyzed by AroA and AroC.

GENSOR units are integrative networks that describe in detail the information flow that goes along the molecular circuitry from signal detection to generation of a response (**Figure 1A**). They formalize the signal-response process in four components. (1) Signal: the molecule that begins the information flow by reflecting a change in the external or internal environment. (2) Signal transduction: the conversion of the signal into a molecule that will prompt a regulator. In the case of TFs, it refers to the conversion of the signal into the effector molecule that binds to the TF. For example, the signal lactose is transformed into allolactose, the molecule that binds to the regulator LacI. (3) Genetic switch: the repression/activation of the specific set of genes needed to contend with the signaled change. (4) Response: the effect of the gene products, which together produce a new phenotype, a change in metabolism, or signal other regulators.

To assemble a GENSOR unit for each of the 189 local TFs, we used a data-driven approach. We automatically retrieved from the RegulonDB database (Gama-Castro et al., 2016) the genes directly regulated by a TF (regulon), its known effectors, its active/inactive conformations, and the TF’s regulatory effect over the regulated genes. From the EcoCyc database (Keseler et al., 2013), we automatically obtained the gene products of the regulated genes, the reactions catalyzed by the gene products, the substrates and products of the catalyzed reactions, and the protein complexes in which gene products participate. It is important to note that the only heuristics included in our method is to include no more genes than those directly regulated by the TF. An exhaustive search is performed to retrieve all the available elements that have been experimentally validated, so all the interactions in GENSOR units have been proved to occur naturally.

According to the Jacob and Monod paradigm, each TF will directly regulate genes that together give rise to a cellular capacity, for example, uptake of lactose as the carbon source, production of osmoprotectants, or flagellar assembly. From this assumption, it follows that the four components of the GENSOR unit can be identified within the retrieved set of elements and their interactions (**Figure 1B**). Eighty GENSOR units included a known effector, and it was possible to identify their four components. The resulting integrative networks described in detail the steps from signal detection to metabolic impact, which were summarized in a short sentence (**Figure 1B**). For the remaining 109 GENSOR units, only genetic switches and response information was pinpointed, reflecting the physiological effect of the TF. It is relevant to note that our previous assumption excluded three occurrences: constitutive enzymes, cooperation between TFs, and yet-unknown regulatory interactions. Any of them could account for genes involved in the biological process depicted by a GENSOR unit, but they are not present because they are not directly regulated by the TF. In order to enrich GENSOR units with known functional interactions, we considered canonical metabolic pathways. Links were added between metabolites already present in a GENSOR unit if they belonged to the same metabolic pathway and the directionality of the pathway permitted metabolic flux between them. For example, TrpR regulates seven enzymes of the 13 enzymes involved in the production of L-tryptophan from D-erythrose-4P (**Figure 1C**). Considering only TrpR’s direct targets, it would appear that the reactions catalyzed by AroH and AroL are not related. However, the metabolic pathways of chorismate biosynthesis from 3-dehydroquinate (**Figure 1C**, purple arrows) and 3-dehydroquinate biosynthesis I (**Figure 1C**, red arrows) indicate the existence of a metabolic flux that converts 3-deoxy-D-arabino-heptulosonate-7P into shikimate and then shikimate-3P into chorismate, respectively. In the TrpR GENSOR unit, the reactions catalyzed by AroB, AroD, and AroE are summarized in one link, termed a secondary reaction, without indication of the involved enzymes or intermediate metabolites (**Figure 1D**, dashed lines). The same happens for reactions catalyzed by AroA and AroC. A total of 144 secondary reactions were added to 48 GENSOR units (Supplementary Figure S2). The 189 GENSOR units are publicly available in the RegulonDB database^1^.

### Feedback Is a Common Occurrence in GENSOR Units

The next step was to identify general properties of GENSOR units. The regulation model by Jacob and Monod (Pardee et al., 1959; Monod et al., 1963) includes the ability of TFs to autotune their activity according to cellular needs and is explained by a direct effect of the regulated response on effector availability. We considered the 80 GENSOR units with known effectors and looked for the presence of reactions that were part of the response while also having a role in the conversion of the signal into the effector. Sixty-five of 78 GENSOR units (83%) included this type of feedback. Two GENSOR units were excluded from the analysis because they did not include regulated enzymes and therefore had no reactions. The simplest feedback consisted of an effector transport through the membrane, and it is interesting that TFs with two or more known effectors had as many feedback loops. The AllS GENSOR unit was the only one whose feedback involved a secondary reaction, suggesting that feedback loops underlie the most basic layer of bacterial decision-making by relying on a single TF switch that senses and responds to these changes. It is possible that feedback loops are a general property of GENSOR units and at least one exists in each, but our method was unable to identify the remaining 17% because they did not rely on metabolic fluxes or use common metabolites that our methodology excludes from the analysis, like DnaA, whose effector is ATP. We expect to identify more feedback loops as new information is included in GENSOR units.

### Complexity of TF Responses Covers a Continuum

We would expect that all of the genes directly regulated by a TF are necessary and sufficient to give rise to a cellular capacity. In fact, bacterial regulators are often referred to as “regulator of X metabolism.” We used the complete set of GENSOR units to obtain a genome-wide distribution of the functional homogeneity of TF responses. In order to exploit the interactions between elements rather than the individual functions of genes, we developed a metric termed connectivity. Connectivity considers the number of individual metabolic fluxes present in a GENSOR unit (and therefore regulated by an individual TF). A metabolic flux is defined as a consecutive set of reactions where the product of a reaction is the reactant of the next one, for example, as in a metabolic pathway (see Materials and Methods). Enzymes that catalyze individual reactions in a metabolic flux are considered “connected,” because we assume that enzymes present in the same metabolic flux will be part of the same functional process. Connectivity is calculated as the ratio of connected enzymes (Ec) to total enzymes (Et). If all enzymes are indeed sufficient and necessary for a functional process, we would expect all the regulated enzymes to be connected, and so we penalize deviations by calculating the total independent metabolic fluxes in the GENSOR unit (MFt) and adding the extra fluxes to the denominator. Hence, connectivity is calculated as:

$$C=\frac{Ec}{Et+\left(MFt-1\right)}$$

**FIGURE 2:**
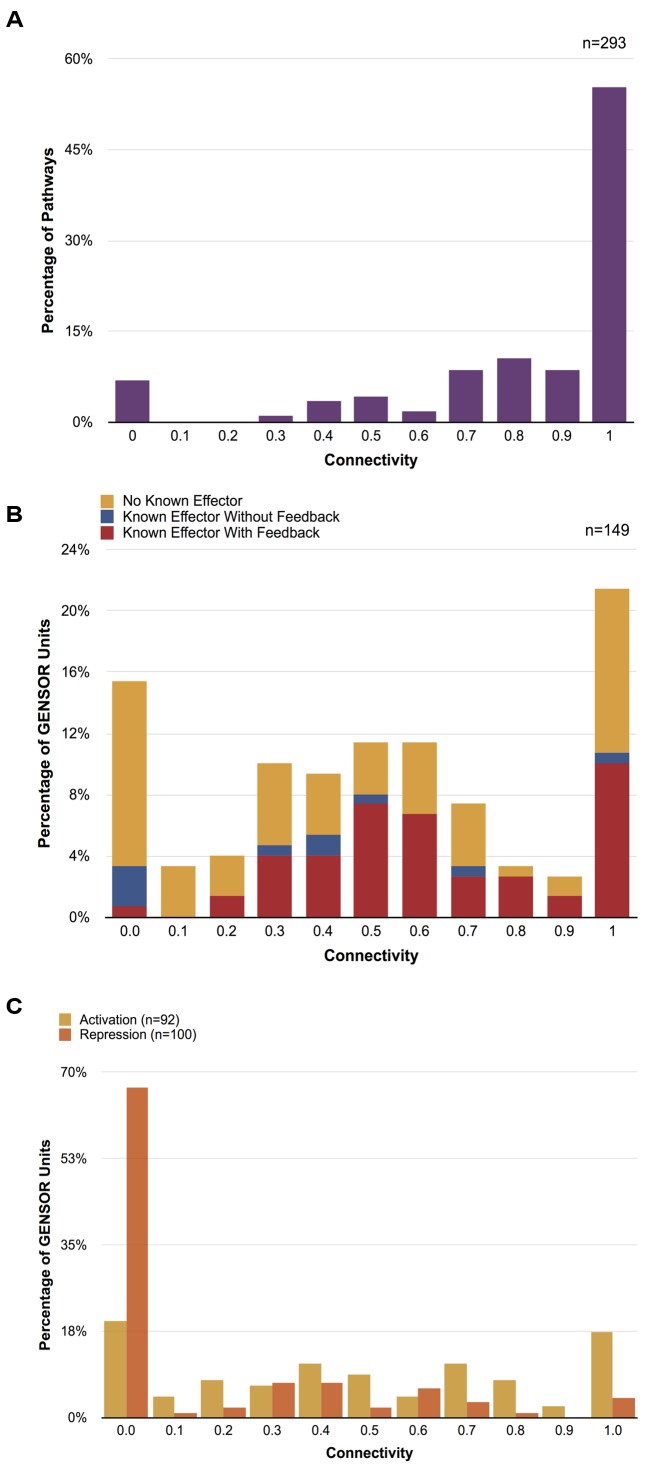
Connectivity distributions. **(A)** Distribution of connectivity values of 293 metabolic pathways. Only sets of genes that catalyze two or more reactions were considered. **(B)** Distribution of connectivity values of 149 GENSOR units. GENSOR units with known effectors and identified feedback loops are shown in red, GENSOR units with known effectors and no identified feedback loops are shown in blue, GENSOR units with no known effectors where only a genetic switch and response have been identified are shown in yellow. The distributions of values in panels **(A)** and **(B)** were significantly different (Wilcoxon–Mann–Whitney; *p*-value < 2.2e-16). **(C)** Distribution of connectivity values of activated (yellow) and repressed (orange) genes in GENSOR units. Only sets of genes that catalyze two or more reactions were considered. Activated and repressed distributions were significantly different (Wilcoxon–Mann–Whitney; *p*-value = 7.724e-11).

Connectivity values range from 0 to 1. A value of 1 indicates a paradigmatic GENSOR unit where all the enzymes are connected and involved in a single metabolic flux. On the other hand, a value of 0 reflects a disconnected topology where each enzyme catalyzes a reaction disconnected from the rest of the GENSOR unit. To validate the biological significance of our metric, we calculated the connectivity of the 293 base pathways reported in EcoCyc (**Figure 2A**). The majority of metabolic pathways (55%) scored a value of 1, and 84% scored 0.7 or higher. A value of 0 was present for pathways such as tRNA charging, in which metabolic reactions are not successive but are functionally related. Results showed that connectivity does reflect a biological property, albeit with an expected 7% margin of error due to pathways that are not linear.

We calculated the connectivity distribution of 149 GENSOR units. Forty were excluded from the analysis because they included less than two catalytic reactions and would produce artificial values of 0. The resulting connectivity distribution (**Figure 2B**) was significantly different (Wilcoxon–Mann–Whitney; *p*-value < 2.2e-16) from the metabolic pathway distribution (**Figure 2A**), which is noteworthy because we enriched the GENSOR unit set to include known metabolic pathways through the addition of secondary reactions. This result showed that the metabolic response mediated by TFs does not correlate with canonical metabolic pathways. The largest proportion of GENSOR units (21%) had a response involved in an individual metabolic flux, including 23% of GENSOR units for which feedback was present. In contrast, in the second largest proportion, 15% of GENSOR units had a connectivity of 0, followed by 11% with values of 0.5 and 0.6. The resulting gradient of connectivity is not likely to be an artifact of unknown binding sites, since it is present in the set of the most extensively studied TFs (TFs with known effectors; **Figure 2B**, red and blue bars), as well as in the set of less-studied TFs (TFs without known effectors; **Figure 2B**, yellow bars). It is important to note that connectivity of a GENSOR unit did not depend on the number of enzymes present in it (Supplementary Figure S3). Moreover, the gradient is still observed if only GENSOR units with five enzymes or less are considered.

The connectivity of GENSOR units in the presence of feedback (**Figure 2B**, red bars) can be interpreted as a measure of autonomy of the TF response. Having a value of 1 means that, in the presence of the signal, the TF will impact a single metabolic flux that has an effect on said signal availability. Therefore, the response will continue until the signal concentration changes. Other forces can act on the metabolic flux, but from the TF perspective its effect is straightforward. A total of 19 TFs fall into this category, including those with responses involved in allantoin (AllS, AllR), arsenite (ArsR), hydroxybutyrate (AtoC), choline (BetI), chitobiose (ChbR), cyanate (CynR), nickel (NikR), zinc (Zur, ZntR), Fe^2+^ (YqjI), acetylneuraminate (NanR), 3-(3-hydroxyphenyl)propanoate (MhpR), idonate (IdnR), glycine (GcvA), citrate (PrpR), gluconate (GntR), tryptophan (TrpR), and xanthosine (XapR) metabolism.

The TF regulatory effect could account for low connectivity values and a higher complexity of a GENSOR unit response. Activation of a metabolic flux needs the presence of all the necessary enzymes, but inhibition of a pathway and redirection of the metabolic flux can be achieved by repressing a single gene. Following this logic, we would expect lower connectivity values for repressed enzymes. To test this hypothesis, we calculated connectivity of activated and repressed genes in each GENSOR unit separately. Consistent with our hypothesis, the connectivity distribution of repressed genes had a peak at 0 that included 67% of tested GENSOR units (**Figure 2C**) and was significantly different from the distribution of activated genes (Wilcoxon–Mann–Whitney; *p*-value = 7.724e-11). GENSOR units with the lowest connectivity values might be regulatory checkpoints that affect several independent metabolic fluxes in response to a stimulus, producing a more global response. Ultimately, connectivity reflects the complexity of the response mediated by a TF and, as we have shown, it is a continuum with peaks on both sides of the scale. Physiologically relevant metrics like connectivity might aid in more accurate functional classifications for regulators.

### Prediction of Effectors Using GENSOR Unit Topology

The value of GENSOR units lies partially in the depiction of the interactions between their elements. They turn lists of regulated genes, enzymes, and reactions into a comprehensive network that reflects the functional effect of a TF. We proceeded to analyze topological properties of the GENSOR units regarding the relationship between effectors and TFs. We considered the set of GENSOR units with known effectors and feedback (**Figure 2B**, red bars), and we identified the position of the effector in the regulated metabolic flux. Effectors were classified into two categories: “substrate/product” if the effector was the first or last metabolite in the metabolic flux, or “intermediate” if it was in any other position. First and last metabolites were grouped in the same category to eliminate ambiguity due to reversible reactions. Ninety-seven percent of the known effectors (75/77) are intermediate metabolites of a metabolic flux (Supplementary Table S1). The high proportion of intermediate effectors is relevant given that only 40% of all metabolites in these GENSOR units with known effectors classify as intermediates under the same criteria. A high proportion of intermediate effectors had been previously observed in inducible catabolic systems (Savageau, 1976). The global analysis presented here suggests that intermediate effectors are a general property, irrespective of the TF mode of action.

It has been shown that using intermediate metabolites as effectors is an effective strategy to increase the stability of a system (Savageau, 1974, 2001). In GENSOR units, stability is crucial to avoid unnecessary production of enzymes under fluctuating signals, which can affect cellular growth rate. Additionally, an intermediate effector will produce two feedback loops. The enzymes upstream of the effector will create a positive feedback loop, where more enzymatic activity will produce more effector. The enzymes downstream of the effector will be involved in a negative feedback loop where the opposite will happen: the more enzymatic activity, the less effector will be present. This dual dynamic produced by intermediate metabolites has been demonstrated by comparing the expression patterns of upstream and downstream enzymes (Chubukov et al., 2012). Accordingly, we observed that upstream and downstream enzymes in GENSOR units tend to be present in different operons. The most frequent case in GENSOR units are effectors positioned as products of transport reactions (here considered within the set of intermediate metabolites), for example, BetI GENSOR unit (**Figure 1B**), and most GENSOR units involved in carbon source utilization and amino acid metabolism. This dynamic might explain why transport enzymes are commonly encoded in a different operon. It is also possible that different dynamics present in the same pathway account for fine-tuning of the metabolic flux in branched pathways, where transport of a metabolite is maximized (through the positive feedback loop), but utilization is modulated so that other pathways can use the metabolite as well. From an evolutionary perspective, an intermediate effector producing two different dynamics would allow the cell to produce more complex metabolic behaviors without the need of new TFs.

The high proportion of intermediate effectors suggested that it would be possible to extrapolate this property to the GENSOR units with no known effector, and to predict effector candidates by retrieving their intermediate molecules. Given the gradient of complexity observed in the GENSOR unit set, it is important to note that the number of effector candidates (and as a result, the number of false positives) will increase according to the complexity of the GENSOR unit in which the method is applied. The median of intermediate metabolites in each GENSOR unit is 11, which is considerably lower than the number of metabolites screened in some heuristic studies (Ibañez et al., 2000). Intuitively, the best predictions would be derived from the simplest GENSOR units, so we applied our method to the 15 GENSOR units with a connectivity value of 1 and no known effector (**Table 1**). Since candidates are taken from the response component of the GENSOR unit, all putative effectors are expected to produce feedback. To support the predictions, experimental evidence was searched in the literature and TFs were searched for ligand-binding domains to further support the mechanism of action.

**Table 1:** Effector predictions in 15 GENSOR units with no reported effector in RegulonDB, and a connectivity value of 1.

GENSOR unit	Ligand binding domain	Predicted effectors	Prediction status	Evidence	Reference
FabR		Fatty acids attached to acyl-ACP	Validated	Gel mobility shift assay	Zhu et al., 2009

UlaR	DeoR C terminal sensor domain (Pfam:PF00455)	Ascorbate-6P	Validated	Gel mobility shift assay	Garces et al., 2008

Dan	Bacterial regulatory helix-turn-helix protein, lysR family (Pfam:PF00126)	Tartrate	Supporting evidence	Change in gene expression due to addition of the compound (β-galactosidase assay)	Kim et al., 2009

FeaR	AraC-binding-like domain (Pfam:PF14525)	Hyacinthin (phenyl acetaldehyde)	Supporting evidence	Inference from operon dynamics	Zeng and Spiro, 2013

HcaR	LysR substrate binding domain (Pfam:PF03466)	3-(5,6-Dihydroxycyclohexa-1,3-dien-1-yl)propanoate	Supporting evidence	Inference from operon dynamics	Turlin et al., 2001

MtlR		Mannitol-1P	Supporting evidence	Inference from operon dynamics	Figge et al., 1994

KdgR	Bacterial transcriptional regulator (Pfam:PF01614)	2-Keto-3-deoxygluconate-6-P	Supporting evidence	2-Keto-3-deoxyguconate has been reported as effector of KdgR ortholog in *Erwinia chrysanthemi*	Nasser et al., 1992

MngR	UTRA domain (Pfam:PF07702)	2 (Alpha-D-mannosyl-6-phosphate)-D-glycerate	Supporting evidence	Change in gene expression due to addition of the external form of the compound (microarray). Mutation of downstream enzymes did not affect induction.	Sampaio et al., 2004

AscG	Periplasmic binding protein-like domain (Pfam:PF13377)	Arbutin-6P, beta-D-cellibiose-6P	New		

CaiF		Gamma-butyrobetaine, crotonobetainyl-CoA, carnityl-CoA, gamma-butyrobetaine-CoA	New. Supported by dynamics of the GENSOR unit (see text). Evidence against other predictions.	Mobility shift assay reflected no binding of L-carnitine or crotonobetaine	Buchet et al., 1999

YiaJ	Bacterial transcriptional regulator (Pfam:PF01614)	Xylulose-5P, 2-3,dioxo-L-gulonate, 3-keto-L-gulonate, 3-keto-L-gulonate 6-P	New. Evidence against other predictions (see text).	80 candidate effectors did not show changes in target gene expression	Ibañez et al., 2000

CsiR	FCD domain (Pfam:PF07729)	L-Glutamate, ketoglutarate, succinate semialdehyde	New		

GatR	DeoR C terminal sensor domain (Pfam:PF00455)	Galactitol 1-phosphate, keto-L-tagatose 6-phosphate, tagatofuranose 1,6-diphosphate	New		

RtcR		RNA terminal-2′,3′-cyclic-phosphate	New		

CadC		Cadaverine, lysine	Evidence against mode of action	Anchored to the membrane; works as a one-component system. Responds to PH stress.	Buchner et al., 2015


*Ligand-binding domains identified in TF sequences are shown alongside their Pfam identifiers. Predicted effectors were inferred from the GENSOR unit topology; the prediction status indicates whether the prediction has been validated in the literature, evidence exists that supports or contradicts the hypotheses, or that predictions are new. ‘Evidence’ column indicates the relevant experiments that have been reported in the literature.*

**FIGURE 3:**
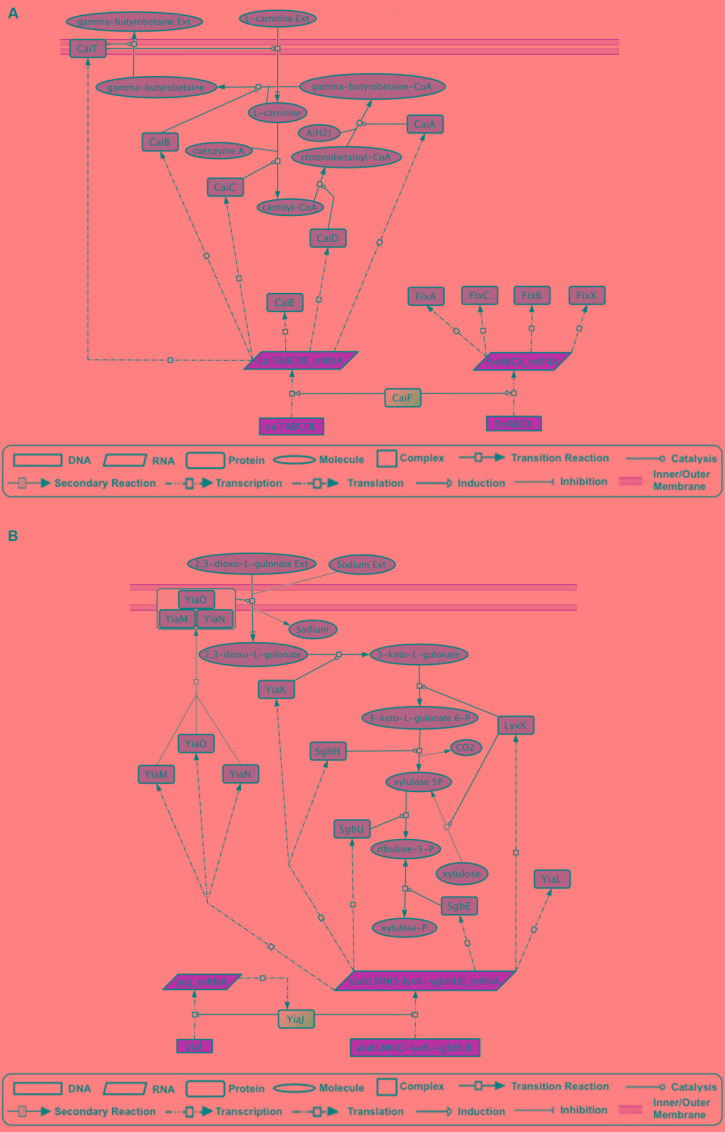
GENSOR unit topologies that allow the inference of hypothetical effectors. **(A)** The CaiF GENSOR unit is active under anaerobic conditions. It is involved in transport of L-carnitine, efflux of gamma-butyrobetaine, and the synthesis of the latter from L-carnitine and gamma-butyrobetaine–CoA. Gamma-butyrobetaine could work as an effector that creates a complex with CaiF and promotes its unbinding from DNA, thus inhibiting the activation of the necessary enzymes for its synthesis and yielding an end product inhibition dynamic that stops the production of the enzymes if the gamma-butyrobetaine concentration inside the cell reaches a threshold. **(B)** The YiaJ GENSOR unit is involved in the transport of 2,3-dioxo-L-gulonate and its transformation into ribulose-5P. The presence of 2,3-dioxo-L-gulonate could promote unbinding of YiaJ from DNA, thus inhibiting the repression of the necessary enzymes for its utilization. Alternatively, xylulose-5P in complex with YiaJ could bind to DNA and promote the inhibition of the necessary enzymes for its synthesis in an end product inhibition dynamic.

Predictions for FabR and UlaR were validated. Supporting evidence was found for hypothetical effectors of Dan, FeaR, HcaR, MtlR, KdgR, and MngR, suggesting that they are excellent candidates for validation experiments. Predictions for AscG, CsiR, GatR, RtcR, CaiF, and YiaJ have not been previously reported in the literature. Evidence for CadC supports a mechanism of action that does not rely on ligand binding (Buchner et al., 2015), which is consistent with its lack of a molecule-binding domain. Two interesting examples of new predictions are CaiF and YiaJ. It has been shown that CaiF acts as an activator in the presence of L-carnitine (Eichler et al., 1996). However, no binding of L-carnitine was identified in a mobility shift assay (Buchet et al., 1999). CaiF GENSOR unit (**Figure 3A**) shows that one of our predictions, gamma-butyrobetaine, would render an end-product inhibition dynamic that fits with the observation of L-carnitine acting as inducer, particularly because it is a substrate in the reaction producing gamma-butyrobetaine. YiaJ negatively regulates the conversion of xylulose and 2,3-dioxo-L-gulonate into D-xylulose 5-phosphate (**Figure 3B**). Ibañez et al. (2000) tested 80 different compounds, including D-xylulose, and none showed a significant increase in expression of target genes. It is possible that our predictions might yield different results, given that they rely on the global interpretation of the interactions in the GENSOR unit. For example, 2,3-dioxo-L-gulonate might act as an effector whose presence unbinds YiaJ from DNA. When bound to DNA, YiaJ would repress the enzymes needed for 2,3-dioxo-L-gulonate utilization as the carbon source, and enzymes would be produced only when it is present in the environment. Since 2,3-dioxo-L-gulonate is converted into D-xylulose 5-phosphate, its uptake would probably only take place when D-xylulose 5-phosphate is not obtained from other carbon sources, like arabinose, xylulose, or ascorbate. Another predicted effector, D-xylulose 5-phosphate, might also act as an effector promoting YiaJ binding to DNA in its presence, rendering an end product inhibition dynamic. We omitted ribulose-5-P from the predictions because it is a central metabolite constantly present in the cell, and the activity of YiaJ has not been reported as central to its metabolism.

In summary, 53% of predictions were supported, 7% were rejected, and 40% have never been reported, including two cases where the GENSOR unit dynamics support the prediction. Because larger data sets are not available, it was not possible to assess our method using receiver operating characteristic curves, but it is important to note that our approach did not require additional tools and it could be used to significantly reduce the search space for possible effectors before experimental procedures.

### GENSOR Units Can Be Used as a Standardized Framework for Integrative Studies

**FIGURE 4:**
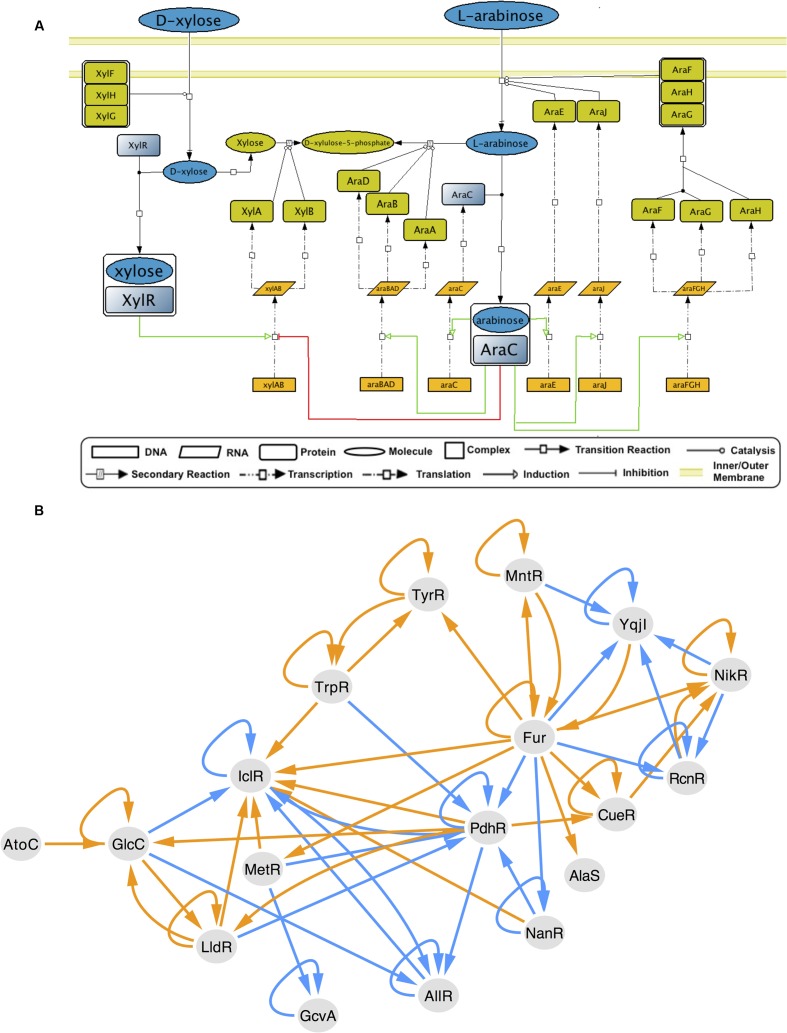
GENSOR unit merging. **(A)** The XylR-AraC complex GENSOR unit. Activation of transcription units is shown in green, and repression is shown in red. Coregulation of *xylAB* with opposite effects creates a switch in carbon source utilization; if arabinose is present and xylose is absent, *xylAB* will be repressed and arabinose will be used as the main carbon source. If xylose is present and arabinose is absent, AraC will stop repressing *xylAB*, XylR will activate it, and xylose will be used. When both carbon sources are present, AraC repression will produce the preferential use of arabinose. **(B)** Cascade of TF–TF regulation through the production of effectors. The cascade is elicited by the activation of the two-component system AtoC in the presence of acetoacetate. AtoC is involved in biosynthesis of polyamines, catabolism of short-chain fatty acids, motility, and chemotaxis and directly regulates only four genes. The metabolic effect of its response produces the effector GlcC, whose response produces other effectors that give rise to a succession of changes in 16 other TF conformations. Edges link TFs that produce an effector in their response to TFs (arrow in edge) that bind to that effector. Orange edges indicate the presence of an effector produces an active conformation in the second TF (arrow in edge), and blue edges indicate inactive conformations.

The collection of regulatory interactions in RegulonDB has been widely used as the gold standard to test new algorithms and as a reliable data set for analysis of the transcriptional regulatory network (TRN). GENSOR units reflect the metabolic impact of that gold standard. Since they were assembled through a data-driven, exhaustive approach, they describe a new layer of biologically relevant knowledge and can be used by the community as a standardized framework for the study of the interplay between transcriptional regulation and metabolism in *E. coli* K-12. One of the main advantages is that GENSOR units are useful for small-scale studies, for example, by analyzing those with high connectivity whose effect is modular. In addition, GENSOR units can be used as building blocks for higher-level descriptions, as high as a whole-cell description that integrates the complete set of GENSOR units through their overlapping elements (Supplementary Figure S4). By merging individual GENSOR units, it is possible to elucidate complex cellular behaviors that involve more than one TF, for example, carbon catabolite repression (Monod, 1942; Görke and Stülke, 2008). When presented with two or more different carbon sources, *E. coli* will begin uptake and utilization in a fixed order: first glucose, then lactose, arabinose, xylose, sorbitol, or rhamnose, and finally ribose (Aidelberg et al., 2014). AraC and XylR are TFs that bind to arabinose and xylose, respectively, and coordinate their utilization. Both regulate the *xylAB* transcription unit, so their GENSOR units can be merged into a complex GENSOR unit (**Figure 4A**). The AraC–XylR complex GENSOR unit shows that when arabinose and xylose are present at the same time, *xylAB* will be repressed by AraC and activated by XylR. Given that in *E. coli* repression tends to be dominant (Collado-Vides et al., 1991), transcription of *xylAB* will be halted and arabinose will be used preferentially. Once arabinose is depleted from the environment, AraC will return to an inactive state. *xylAB* can be induced by XylR, and xylulose will be used as the second carbon source. In summary, the opposite regulation of *xylAB* is the switch where *E. coli* decides on the uptake of arabinose over xylose. AraC–XylR complex GENSOR unit shows the descriptive power of merging individual GENSOR units. More complex decisions can involve more than two GENSOR units, but regardless of the size they retain the same level of detail. Availability of gold standard data sets is a current limitation for studies that integrate regulation and metabolism (Imam et al., 2015). The GENSOR units presented here seek to fill that gap and can also be used for dynamic modeling or analysis of general properties from the complete set of interactions.

Using GENSOR units as building blocks can also shed light onto indirect regulatory mechanisms that rely on metabolism to tune the activity of a TF in the presence of a signal sensed by another TF. The presence of feedback in a GENSOR unit conveys that the response mediated by a TF has a direct effect on its effector availability. Nevertheless, the response can also act on the availability of the effector of a second TF. For example, AlaS binds to L-alanine to solely repress its own promoter; since no other TFs regulate its transcription, it appears as an isolated node in the TRN. However, IscR, NsrR, Fur, and OxyR GENSOR units include the production of alanine in their response through the action of SufS, an L-cysteine desulfurase. In the presence of iron–sulfur clusters, nitric oxide, iron, or oxidative stress (IscR, NsrR, Fur, and OxyR signals), the cellular concentration of alanine will fluctuate, and in turn the AlaS functional conformation will be affected. This rationale can be applied on a larger scale to identify cascades of indirect TF–TF regulation (**Figure 4B**). It will be interesting to couple metabolic and transcriptional regulation cascades, since conformational changes are rarely considered in large-scale analyses of the TRN.

## Discussion

During their lifetimes, cells are challenged with a plethora of perturbations from the environment and their internal machinery. To survive, they rely on genetic circuits that sense changes and give an appropriate response. Understanding how the cell processes information is crucial for advancing the elucidation of design principles. The GENSOR unit concept presented here integrates relationships between the signaling, regulatory, and metabolic networks to depict the information flow behind individual signal → response processes. We have performed a genome-scale analysis of the complexity of the response mediated by each TF, testing the paradigm set by Jacob and Monod (1961) with the regulation of the *lac* operon, whereby regulators work as on/off switches for a particular capacity. Our results showed that, at the genome scale, the relationship between regulated genes and metabolism does not follow an evident one TF/one process rule (**Figures 2A,B**), but design principles can still be observed (**Figure 2C**).

GOs and metabolic pathways are the most used concepts to describe functional units in bacteria. They are mostly used for obtaining functional overviews of groups of genes. However, their definitions of where a process begins and ends are sometimes based on historical and organizational reasoning (such as the presence of common metabolites). Interpretations derived from them are not likely to reflect the way bacteria “understand” function (Bordbar et al., 2014). As an example, 25% of GENSOR units do not include any genes present in a canonical pathway. As for GO enrichment analysis, enriched GO terms in individual GENSOR units range from 0 to 228 (Supplementary Figure S5). Forty percent of GENSOR units have more than 50 enriched GO terms. The hierarchical tree structure of the ontology makes difficult the interpretation of such data, since researchers have to guess to what extent parent–child terms can be thought of as being the same process. Clusters of Orthologous Groups functional categories are also widely used to describe gene function. Given the broadness of the functional terms, they are complementary to GENSOR units. They could be used as a guide to merge individual GENSOR units and describe higher level interactions between their elements. GENSOR units are a stepping stone toward building a framework that integrates transport, signal transduction, gene regulation and metabolism, in a directional regulated process that should capture the logics of information flow as it happens in the cell. They make it possible to trace relationships between genes of interest in their transport, signaling, metabolic, and regulatory contexts at the same time. GENSOR units can address questions such as “Is a group of genes responding to the same signal?” As a conceptual tool, they aim at facilitating the task of making biological sense of high-throughput expression data by reflecting the way that the cell is interpreting the changes that it is being subjected to.

The conceptual integration presented here has opened new questions. For instance, the paradigmatic functional homogeneity among regulons is not a general property. Connectivity values yield a gradient that goes from monothematic to epistatic GENSOR units. It is noteworthy that most GENSOR units are on opposite sides of the connectivity gradient. We propose three non-exclusive hypotheses to explain this. (1) Differences in regulatory mechanisms might produce differences in response properties. For example, two-component systems might account for global responses with low connectivity. (2) Some cellular functions require a more coordinated response. Nutrient uptake and efflux of toxins need fast responses, but flagellum assembly or cell cycle are capacities that depend on the presence of a large set of stable signals. (3) TFs regulate different subgroups of genes under different conditions, creating condition-specific subunits within GENSOR units. Higher-connectivity GENSOR units might pool independent metabolic fluxes that become active under different conditions. This shows the challenge ahead of making sense of the cell circuitry at higher levels of integration.

It has been proposed that coordination of multiple signals is embedded in a series of nested loops where general signals control several modules and local signals control the metabolic flux within modules (Chubukov et al., 2014). In this scenario, feedback loops would be a common occurrence. Small, local loops are for simple and fast responses, like carbon uptake, and larger loops are for central responses, like cell division. The latter requires a combination of signals, encompassed in smaller loops, to be present at a given time. This hypothesis agrees with our observations of GENSOR units. (1) Feedback loops are a common occurrence. (2) There is a gradient of modularity in GENSOR units reflected by connectivity values, from evident self-sufficient topologies to unrelated reactions that need the presence of other GENSOR units to be interpreted. (3) GENSOR units can be linked through “reporter molecules,” such as effectors that can signal to general regulatory programs (**Figure 4B**). (4) GENSOR units can be merged to create a broader feedback loop (Supplementary Figure S6).

To the best of our knowledge, there is no other framework that comprises the complete catalog of natural genetic circuits mediated by TFs. Since GENSOR units place regulation in its natural cellular context, the concept is in line with operons and regulons and can be interpreted as a higher-level natural unit. GENSOR units also provide twofold methodological novelty. First, there is the automatic integration of data through an exhaustive search that eliminates any bias toward what the curator knows about the regulator. For example, rarely is it mentioned in descriptions of the *lac* operon that LacY can also transport melibiose. The only inherent bias is the availability of data in the two databases used. However, GENSOR units could be updated, as for any data set, with each new database release. The second methodological novelty is the prediction of effectors from the topology of the GENSOR unit alone. Although further validations are needed, we have proved that the information provided by GENSOR units can meaningfully reduce the number of effector candidates before designing an experiment.

**FIGURE 5:**
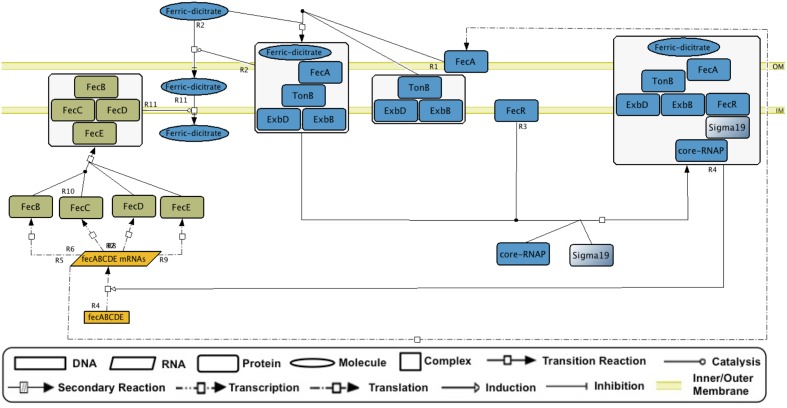
The sigma 19 (FecI) GENSOR unit. In the presence of ferric dicitrate, FecI activates transcription of genes that code for proteins involved in transport of ferric dicitrate. Events occur as follows: external ferric dicitrate binds to FecA and causes a conformational change that allows FecA binding to TonB. FecA, TonB, and ExbD, and ExbB forms a complex that transports external ferric dicitrate into the periplasmic space. Alternatively, this complex binds to FecR, a protein that binds to sigma 19 and stimulates the binding of sigma 19 to RNA polymerase. Finally, the complex stimulates the transcription of the genes that code for proteins involved in the transport of ferric dicitrate from the periplasmic space to the cytoplasm. The signal and signal transduction is shown in blue, the genetic switch is shown in yellow, and the response is shown in green.

GENSOR units can be used as templates for dynamic modeling. Either individually or merged to trace metabolic fluxes of interest, the GENSOR units can be used to predict the effects of adding molecules in the medium and to identify functional modules. They can also be used as a gold standard for new methodologies that predict properties of the interplay between transcriptional regulation and metabolism. Efforts are currently being made to assemble GENSOR units for other bacteria. It will be interesting to compare rewiring of their components, considering that TF binding sites diverge faster than coding sequences (Borneman et al., 2007). It might be possible to identify “orthologous” topologies that produce the same functional output using different network architectures. Identifying GENSOR units in pathogenic strains could also help in antibiotic design. Finally, the conceptual framework of GENSOR units can be expanded to other types of regulators. We have assembled a GENSOR unit of sigma factor 19 from *E. coli* that shows the four components and a feedback loop (**Figure 5**). Eventually, GENSOR units could be applied to eukaryotic regulators involved in disease to understand the mechanisms that cause disruptions in cellular dynamics, for example, the disappearance of feedback loops.

## Materials and Methods

### GENSOR Unit Assembly

Active and inactive conformations, effectors, and regulated genes of the 189 local TFs with experimental evidence for their regulatory activities were obtained automatically from RegulonDB data sets; the Perlcyc API of Pathway Tools was used for automatic retrieval of gene products, catalyzed reactions, substrates, products, and directionality of reactions, heteromultimeric protein complexes in which gene products participate, and the rest of the monomers involved in the complex. Data were used to automatically generate an SBML file using CellDesigner 4.4 (Funahashi et al., 2008), the resulting network that was manually inspected and components in GENSOR units were identified. Secondary reactions were included by identifying pairs of reactions in the GENSOR unit that belonged to the same metabolic pathway using Perlcyc API of Pathway Tools software, the connecting reactions were added as a single secondary reaction only if directionality was maintained. Reversible reactions were considered. The resulting network and the information on each element and interaction were added to RegulonDB.

### Properties of GENSOR Units

All properties were automatically identified and/or quantified using custom perl scripts available at GitHub^2^.

#### Metabolic Fluxes in GENSOR Units

A metabolic flux was considered a chemical transformation of one metabolite into another; it could comprise one or more enzymatic reactions. A metabolic flux of two or more enzymatic reactions was created when substrates of one reaction were present in another either as substrates or products. Directionality of the reactions was considered to infer directionality of metabolic flux.

#### Feedback

All the possible metabolic fluxes in each GENSOR unit were obtained. Feedback was considered present when an effector was involved in one or more metabolic fluxes. Two-component systems were not considered, because their effector molecule was annotated as phosphate.

#### Connectivity

Connectivity was calculated through the following formula:

$$C=\frac{Ec}{Et+\left(MFt-1\right)}$$

where Ec is the connected enzymes in the GENSOR unit; Et is the total enzymes in the GENSOR unit; and MFt is the total independent metabolic fluxes in the GENSOR unit. Two enzymes were connected if any of their catalyzed reactions were also connected. Two reactions were connected if they shared substrates or the product of a reaction was also the reactant of a second reaction. Metabolic fluxes are independent groups of connected reactions (by the criteria described above), e.g., two components were present in a GENSOR unit if two groups of reactions were not connected between them but reactions within each group created a continuous flux.

### Connectivity of Metabolic Pathways

Canonical pathways and the genes involved in each were obtained from EcoCyc by using the Pathway Tools software, Perlcyc API, and custom Perl scripts. A total of 362 base pathways were introduced to a modified version of the GENSOR unit pipeline; only 293 included two or more enzymatic reactions, and connectivity was calculated for these. Regulation of the genes was not considered. The pipeline used for the analysis can be found at GitHub^3^.

### Connectivity of GENSOR Units, Considering Their Regulatory Effects

Metabolic fluxes identified in each GENSOR unit only considered reactions catalyzed by enzymes whose genes were subject to the same type of regulation (activation/repression). Dual and unknown regulatory interactions were considered in both sets. Connectivity was obtained with the same algorithm, considering activated and repressed metabolic fluxes separately. The pipeline used for the analysis can be found at GitHub^4^.

### Effector Predictions

#### Position of Effector in Pathway

Seventy-eight GENSOR units with known effectors were analyzed. The position of each effector in the regulated pathway was automatically retrieved using a custom Perl script. The classification criteria were as follows:

- Substrate/product. Effectors that had a role as reactant in only one enzymatic reaction in the GENSOR unit. First and last positions were grouped to decrease ambiguity due to reversible reactions.

- Intermediate. Effectors that had a role as reactant in two or more enzymatic reactions in the GENSOR unit. Effectors that were products of transport reactions were considered intermediates to follow the classification defined by Savageau (1976).

#### Selection of Effector Candidates

GENSOR units with a connectivity value of 1 and no reported effectors in RegulonDB were used to predict effectors from their topology. All metabolites in the GENSOR unit were classified as substrate/product or intermediate according to the criteria mentioned above. Resulting intermediate metabolites were searched in the literature for experimental evidence of ligand function. The metabolites with supporting evidence on each GENSOR unit were considered hypothetical effectors; if no information was available for any of the molecules, all were reported as new candidates. Ligand-binding domains of the 15 TFs were identified using the NCBI Conserved Domains and Pfam (Finn et al., 2016) databases.

### Statistical Analyses

Wilcoxon–Mann–Whitney tests were performed through a Wilcoxon rank sum test with continuity correction, using R software.

### AtoC Cascade of Indirect TF Regulation

GENSOR units that included a reaction producing the effector of their own reaction of or another GENSOR unit were identified using custom Perl scripts on the relational tables of the GENSOR unit data set. The AtoC cascade was identified manually using AtoC as the starting point; all GENSOR units whose TFs bound to a metabolite produced in the AtoC GENSOR unit were included in the cascade and used as new starting points. The algorithm was run recursively until no more effectors were present in the lowest-level GENSOR unit response. The cascade network was produced using Cytoscape v3.1.1 (Shannon et al., 2003).

### Metabolic Pathways and Gene Ontologies in GENSOR Units

Metabolic pathways of all genes were obtained from EcoCyc using Pathway Tools software. GOs of all genes were obtained from the Gene Ontology Consortium^5^. GO enrichments were obtained using the SmartTables tool in EcoCyc. Enrichments were calculated using all the genes in a GENSOR unit, along with analysis via the Fisher exact statistic with Bonferroni correction; *p*-values of <0.05 were considered statistically significant. A pathway/GO term was considered “present” in all the GENSOR units that included at least one gene from the pathway/GO term.

### Data and Code Availability

GENSOR units in network form are publicly available in RegulonDB^6^, and GENSOR units in tab-delimited files are publicly available at GitHub^7^. All scripts and files generated in the analysis are also available at GitHub^8^.

## Author Contributions

JC-V conceived, designed, and supervised the study, and critically edited the manuscript. DL-T designed the study, performed all computational analysis, and wrote the manuscript. CI performed all manual curation, validated and summarized the GENSOR units, and wrote the manuscript. All authors read and approved the final manuscript.

## Conflict of Interest Statement

The authors declare that the research was conducted in the absence of any commercial or financial relationships that could be construed as a potential conflict of interest.

## Footnotes

1 http://regulondb.ccg.unam.mx/

2 https://github.com/dledezma/gensor_units/tree/master/perl_scripts

3 https://github.com/dledezma/gensor_units/blob/master/pipelines/pipeline_connectivity_pathways.sh

4 https://github.com/dledezma/gensor_units/blob/master/pipelines/pipeline_connectivity_with_effect.sh

5 http://geneontology.org/page/download-annotations

6 http://regulondb.ccg.unam.mx/central_panel_menu/integrated_views_and_tools/gensor_unit_groups

7 https://github.com/dledezma/gensor_units/tree/master/datasets

8 https://github.com/dledezma/gensor_units/

## Abbreviations

CCR: carbon catabolite repression
GENSOR unit: genetic sensory response unit
TF: transcription factor
TRN: transcriptional regulatory network.
