# Supplementary material for: Genome-Wide Mapping of Transcriptional Regulation and Metabolism Describes Information-Processing Units in Escherichia coli
Source: Front Microbiol. 2017 Aug 3;8:1466. doi: 10.3389/fmicb.2017.01466 (PMC5540944; doi:10.3389/fmicb.2017.01466)
Supplement: Supplementary file 1 [file Table_1.pdf]

| GU name | Effector | Effector Position |
|---|---|---|
| AlaS | alanine | intermediate |
| AllR | glyoxylate | intermediate |
| AllS | allantoin | intermediate |
| AlsR | allose | intermediate |
| AraC | arabinose | intermediate |
| ArgP | arginine | intermediate |
| ArgP | lysine | intermediate |
| ArgR | arginine | intermediate |
| ArsR | arsenite | intermediate |
| AsnC | asparagine | intermediate |
| BetI | choline | intermediate |
| ChbR | N-monoacetylchitobiose 6'-phosphate | intermediate |
| Cra | fructose 1,6-biphosphate | intermediate |
| Cra | fructose 1-phosphate | intermediate |
| CueR | Cu(I) | intermediate |
| CynR | cyanate | intermediate |
| CysB | acetylserine | intermediate |
| CysB | sulfide | intermediate |
| CysB | thiosulfate | intermediate |
| CytR | cytidine | intermediate |
| DeoR | 2-deoxy-D-ribose 5-phosphate | intermediate |
| DhaR | DhaK | intermediate |
| DsdC | serine | intermediate |
| FhlA | formate | intermediate |
| FucR | fuculose-1-P | intermediate |
| Fur | Fe+2 | intermediate |
| Fur | Mn(II) | intermediate |
| GalR | galactose | intermediate |
| GalS | galactose | intermediate |
| GcvA | glycine | intermediate |
| GlcC | glycolate | intermediate |
| GlpR | glycerol-3-phosphate | intermediate |
| GntR | gluconate | intermediate |
| GutR | gulitol | substrate /product |
| HipB | HipA | intermediate |
| IclR | glyoxylate | intermediate |
| IdnR | 5-ketogluconate | intermediate |
| IdnR | idonate | intermediate |
| IlvY | alpha-acetolactate | intermediate |
| LacI | allolactose | intermediate |
| LldR | lactate | intermediate |
| LsrR | AI-2 | intermediate |
| MalT | MalK | intermediate |
| MalT | maltotriose | intermediate |
| MelR | melibiose | intermediate |
| MetJ | SAM | intermediate |
| MetR | homo-cys | intermediate |
| MhpR | 2,3-DHP | intermediate |
| MhpR | 3HPP | intermediate |
| MntR | Mn(II) | intermediate |
| ModE | molybdate | intermediate |
| MqsA | MqsR | intermediate |
| MurR | MurNAc-6-P | intermediate |
| NanR | N-acetylneuraminate | intermediate |
| NhaR | Sodium | intermediate |
| NikR | nickel | intermediate |
| NrdR | dATP | intermediate |
| PdhR | pyruvate | intermediate |
| PrpR | (2S,3S)-2-methylcitrate | intermediate |
| RcnR | cobalt ion | intermediate |
| RcnR | nickel | intermediate |
| RelB | RelE | intermediate |
| RhaS | rhamnose | intermediate |
| RutR | thymine | intermediate |
| RutR | uracil | intermediate |
| TreR | alpha,alpha-trehalose 6-phosphate | intermediate |
| TreR | trehalose | substrate /product |
| TrpR | tryptophan | intermediate |
| TyrR | phenylalanine | intermediate |
| TyrR | tryptophan | intermediate |
| TyrR | tyrosine | intermediate |
| UxuR | fructuronate | intermediate |
| XapR | xanthosine | intermediate |
| XylR | xylose | intermediate |
| YqjI | Fe+2 | intermediate |
| ZntR | Zinc | intermediate |
| Zur | Zinc | intermediate |

**Table S1**. Position of effectors with feedback in the regulated pathway of their GENSOR Units.
