# Supplementary material for: Genome-Wide Mapping of Transcriptional Regulation and Metabolism Describes Information-Processing Units in Escherichia coli
Source: Front Microbiol. 2017 Aug 3;8:1466. doi: 10.3389/fmicb.2017.01466 (PMC5540944; doi:10.3389/fmicb.2017.01466)
Supplement: Supplementary file 2 [file Image_1.PDF]

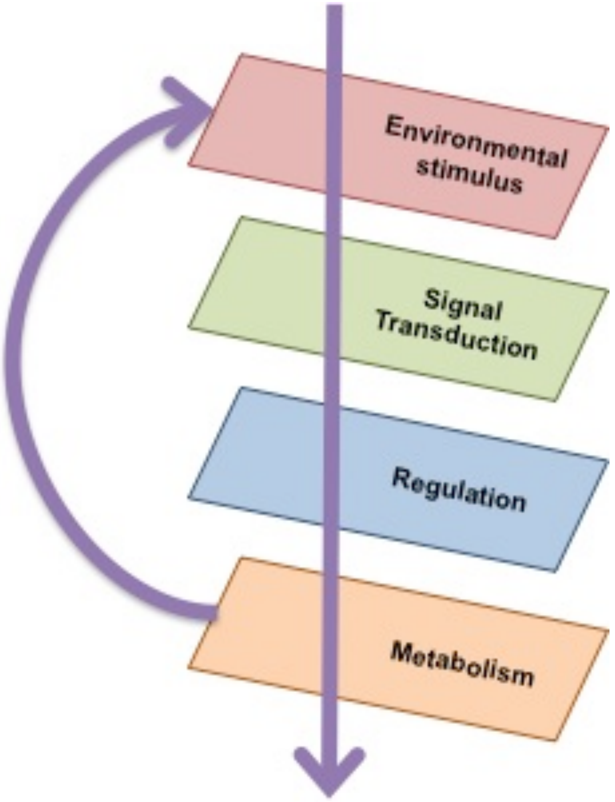

Figure S1. Integration of cellular layers. GENSOR units describe the flow of information across the signal transduction pathways, the transcriptional regulatory network, and the metabolic network. GENSOR Units are guided by the effects of individual regulators and link them to the signal responsible for their effect on gene expression.
