# Supplementary material for: Genome-Wide Mapping of Transcriptional Regulation and Metabolism Describes Information-Processing Units in Escherichia coli
Source: Front Microbiol. 2017 Aug 3;8:1466. doi: 10.3389/fmicb.2017.01466 (PMC5540944; doi:10.3389/fmicb.2017.01466)
Supplement: Supplementary file 3 [file Image_2.pdf]

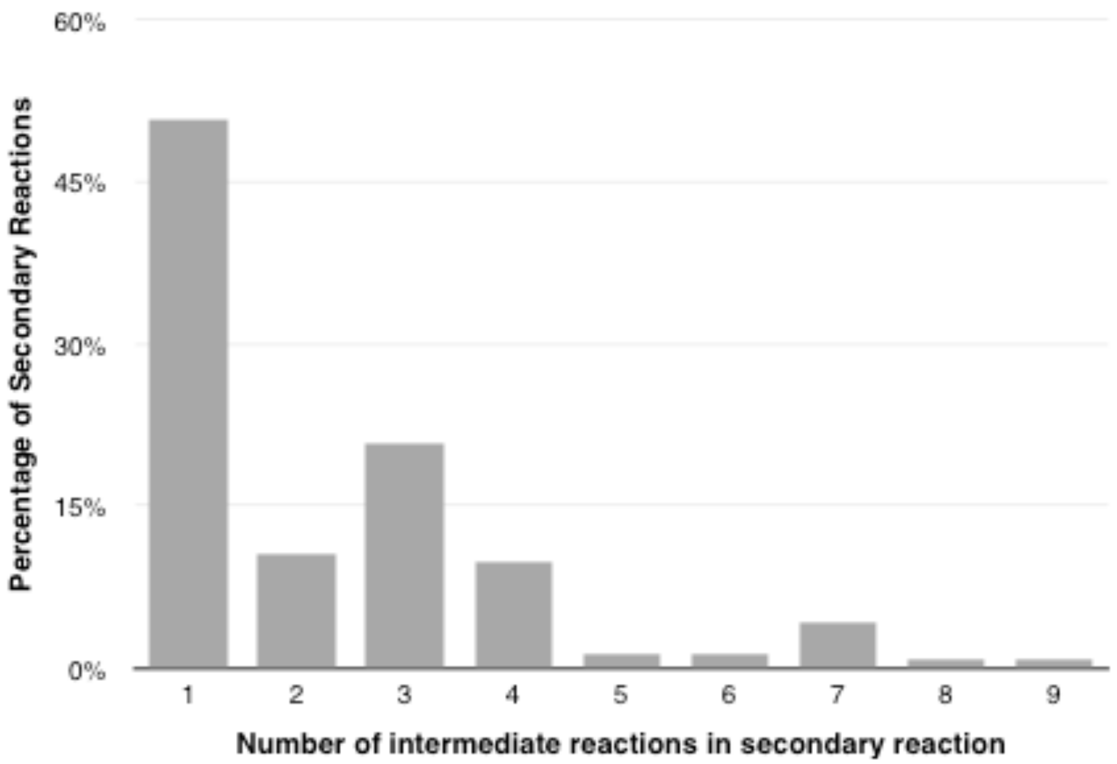

Figure S2. Distribution of individual reactions in secondary reactions. As seen in Figure 1C-D, each secondary reaction represents one or more intermediate reactions in a metabolic pathway that connects two metabolites in a GENSOR unit. A total of 144 secondary reactions were added to 48 GENSOR units. The x axis shows the number of intermediate reactions. The y axis shows the percentage of secondary reactions added via x number of intermediate reactions. Almost 50% of secondary reactions included only one intermediate reaction.
