# Supplementary material for: Genome-Wide Mapping of Transcriptional Regulation and Metabolism Describes Information-Processing Units in Escherichia coli
Source: Front Microbiol. 2017 Aug 3;8:1466. doi: 10.3389/fmicb.2017.01466 (PMC5540944; doi:10.3389/fmicb.2017.01466)
Supplement: Supplementary file 4 [file Image_3.PDF]

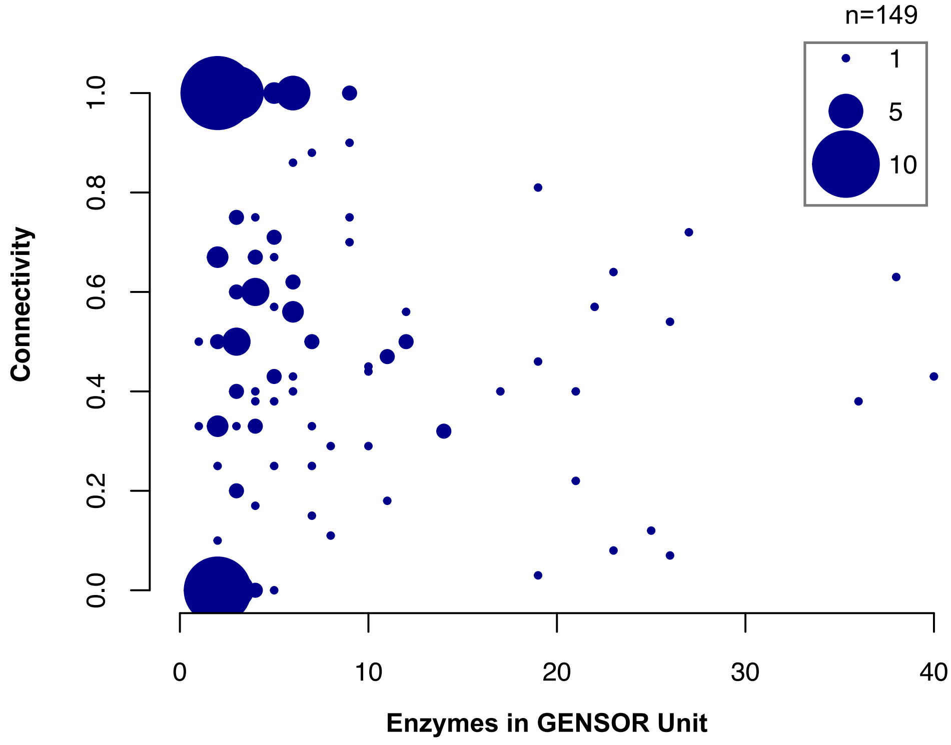

Figure S3. Total enzymes in a GENSOR Unit compared to its connectivity value. Only enzymes where the catalyzed reaction is known were considered. GENSOR Units with less than two catalytic reactions were omitted to avoid values of 0 without biological significance.
