# Supplementary material for: Genome-Wide Mapping of Transcriptional Regulation and Metabolism Describes Information-Processing Units in Escherichia coli
Source: Front Microbiol. 2017 Aug 3;8:1466. doi: 10.3389/fmicb.2017.01466 (PMC5540944; doi:10.3389/fmicb.2017.01466)
Supplement: Supplementary file 5 [file Image_4.PDF]

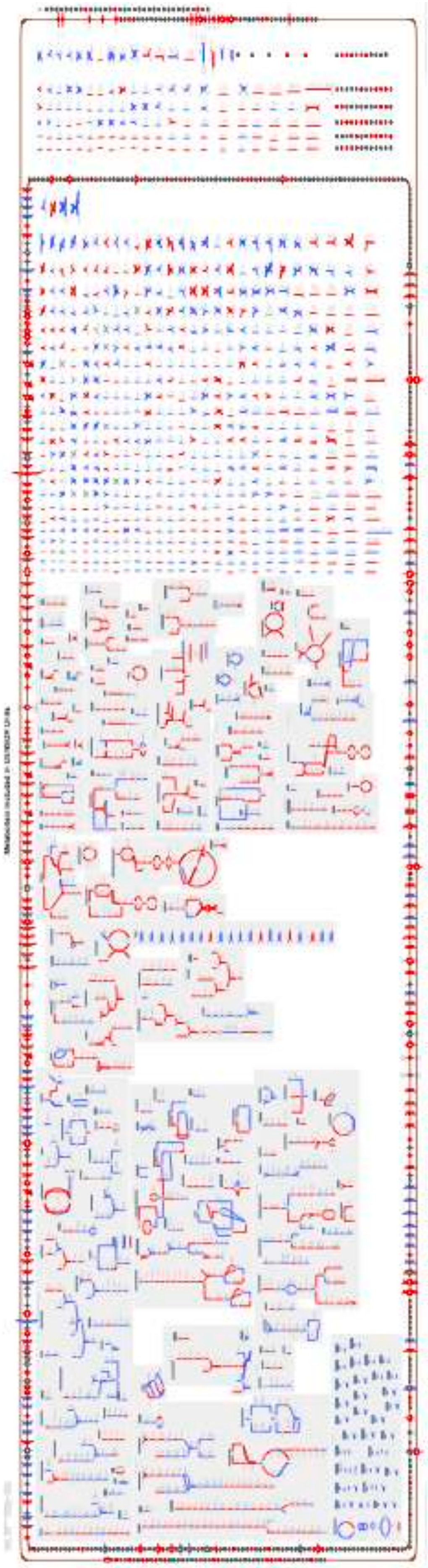

Figure S4. Fraction of metabolism covered by GENSOR units. Cellular overview from EcoCyc showing in red the reactions included in the GENSOR unit collection. (https://ecocyc.org/overviewsWeb/celOv.shtml). Most metabolic pathways are covered, especially those for carbon, amino acid, and lipid metabolism. An SBML map of the 189 merged GENSOR units is available on demand.
