# Supplementary material for: Genome-Wide Mapping of Transcriptional Regulation and Metabolism Describes Information-Processing Units in Escherichia coli
Source: Front Microbiol. 2017 Aug 3;8:1466. doi: 10.3389/fmicb.2017.01466 (PMC5540944; doi:10.3389/fmicb.2017.01466)
Supplement: Supplementary file 6 [file Image_5.pdf]

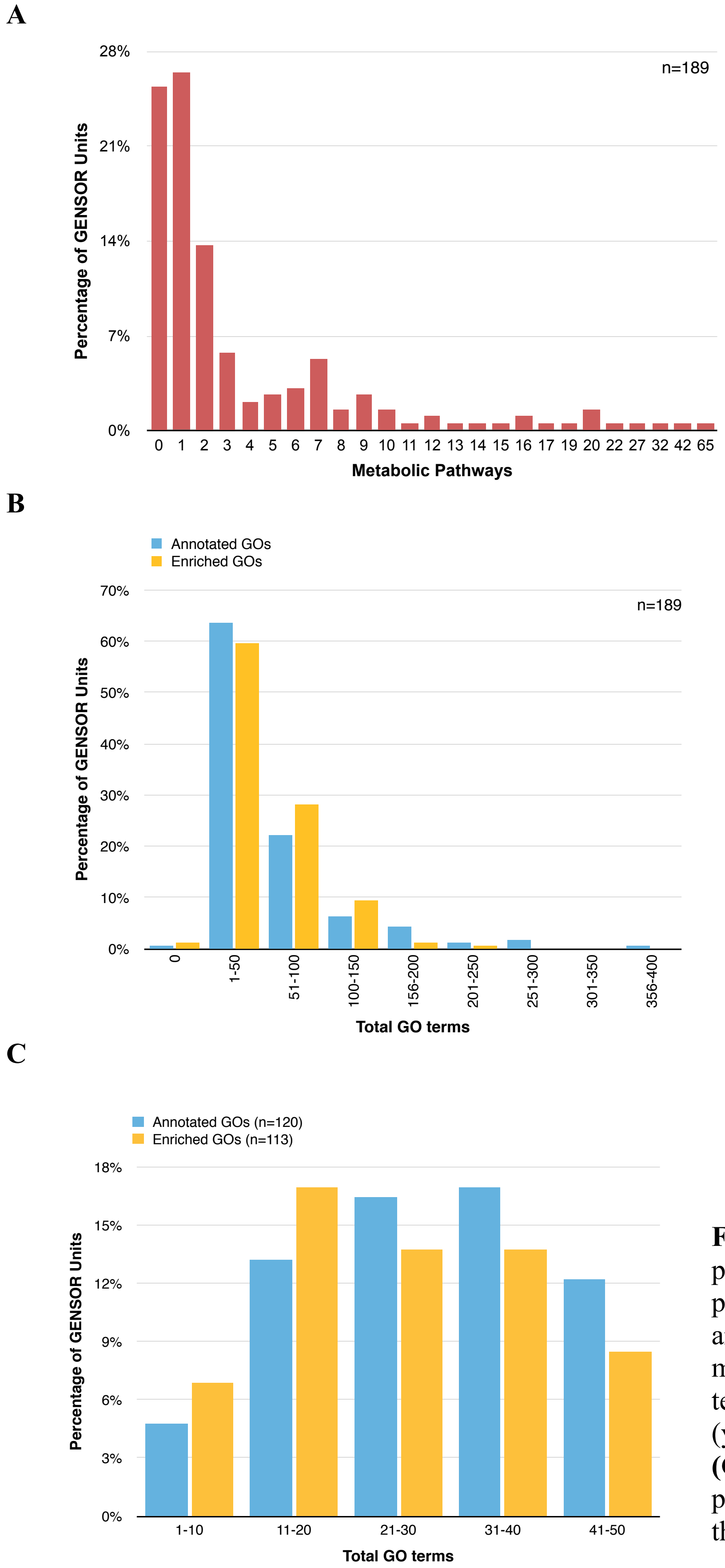

Figure S5. Distribution of metabolic pathways and gene ontology terms present in the 189 GENSOR units analyzed. **(A)** Distribution of total metabolic pathways. **(B)** Total GO terms present (blue) and enriched (yellow) in the 189 GENSOR units. **(C)** Distribution of the 1-50 group from panel B. Color coding is the same as that described for panel B.
