# Supplementary material for: Genome-Wide Mapping of Transcriptional Regulation and Metabolism Describes Information-Processing Units in Escherichia coli
Source: Front Microbiol. 2017 Aug 3;8:1466. doi: 10.3389/fmicb.2017.01466 (PMC5540944; doi:10.3389/fmicb.2017.01466)
Supplement: Supplementary file 7 [file Image_6.PDF]

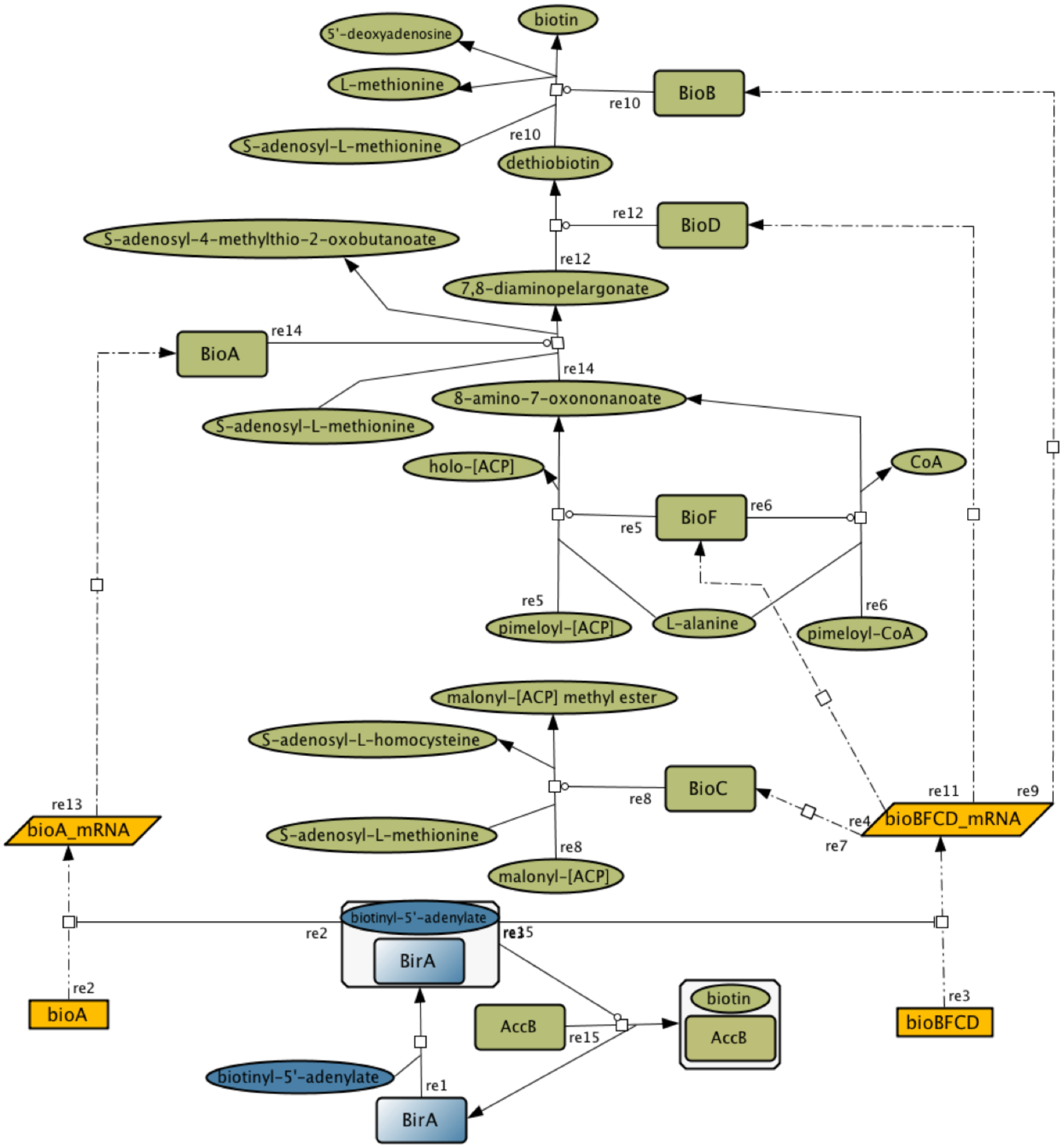

Figure S6. BirA and AccB interactions form a feedback loop that includes two TFs. BirA regulates biotin synthesis, and its active conformation, BirA-bio-5'-AMP, promotes AccB-biotin complex formation, which in turn returns BirA to its inactive state.
